# The Prospect of Non-Alcoholic Fatty Liver Disease in Adult Patients with Metabolic Syndrome: A Systematic Review

**DOI:** 10.7759/cureus.41959

**Published:** 2023-07-16

**Authors:** Zareen Zohara, Ademiniyi Adelekun, Kofi D Seffah, Korlos Salib, Lana Dardari, Maher Taha, Purva Dahat, Stacy Toriola, Travis Satnarine, Ana P Arcia Franchini

**Affiliations:** 1 Research, California Institute of Behavioral Neurosciences & Psychology, Fairfield, USA

**Keywords:** dyslipidemia, type 2 diabetes, obesity, insulin resistance, syndrome x, metabolic syndrome

## Abstract

In recent years, there has been an increasing trend in the development of non-alcoholic fatty liver disease (NAFLD) due to lifestyle changes. The limited treatment option for the disease makes it challenging to manage. This study aims to summarize the relationship between NAFLD and metabolic syndrome (MetS) and to give a clear idea of the risk factors in this systematic research. The five databases screened were PubMed, Google Scholar, Science Direct, and BMC using keywords and Medical Subject Heading (Mesh) combinations. The keywords used are “Metabolic Syndrome,” “Syndrome X,” “Insulin Resistance,” “Obesity,” “Type 2 Diabetes,” and “Dyslipidemia.” Articles underwent a detailed process of screening and quality appraisal. Using the English language as a primary filtering parameter, papers over the last 13 years, dating from 2010 to 2023, are the basis of this review. We reviewed all possible human studies documenting NAFLD with a component of MetS. A total of 1106 papers were identified. After duplicate removal, 995 articles underwent a rigorous review, and 35 articles were chosen for quality appraisal. A total of 15 articles are part of this systematic review. This systematic review strongly concludes that NAFLD predominates in MetS patients. The pathophysiology and insulin resistance that is shared by the two conditions as well as the fact that obesity is at the center of both is the connecting factor in this. Besides various demographic and risk factors, physical activity and diet also play a role in the development of NAFLD. Consequently, more studies on this relevant topic are needed.

## Introduction and background

Non-alcoholic fatty liver disease (NAFLD) is a catch-all term for a variety of conditions when steatosis is present in more than 5% of hepatocytes and there are metabolic risk factors (particularly type 2 diabetes and obesity), excluding excessive alcohol intake or other chronic liver illnesses [1]. The term "steatotic liver disease" (SLD) was used to cover all of the many causes of steatosis. The name "steatohepatitis" was believed to represent an essential pathologic idea that ought to be preserved. Metabolic dysfunction-associated steatosis liver disease (MASLD) was chosen to replace NAFLD. Non-alcoholic fatty liver and its more advanced form, non-alcoholic steatohepatitis (NASH), have attracted much attention because of their expanding influence on global health due to the changing lifestyle. NAFLD instances are expected to increase in the United States from 83.1 million in 2015 (almost 25% of the population) to 100.9 million in 2030 [2]. The term "NAFLD" is an umbrella term for a range of non-fibrotic and fibrotic liver conditions. The risk of negative outcomes is extremely low in individuals with just NAFLD, although patients with just NAFLD had a reduced risk of issues with their liver and other organs than those with NASH. Adverse hepatic outcomes of NASH are cirrhosis, liver failure, and hepatocellular carcinoma [3,4]. NAFLD is the most prevalent cause of abnormal liver function tests and is currently more common than alcoholic liver disease due to a sharp increase in obesity prevalence, even though only a minority of NAFLD patients will experience problems from their chronic liver disease. The overall number of NAFLD-related end-stage liver disease patients is rapidly increasing; from 2004 to 2013, there was a 170% increase in the number of NASH cases in the United States on the transplant waiting list. NASH is expected to overtake other liver diseases as the main reason for liver transplantation in the United States in the upcoming years [1]. 

**Figure 1:**
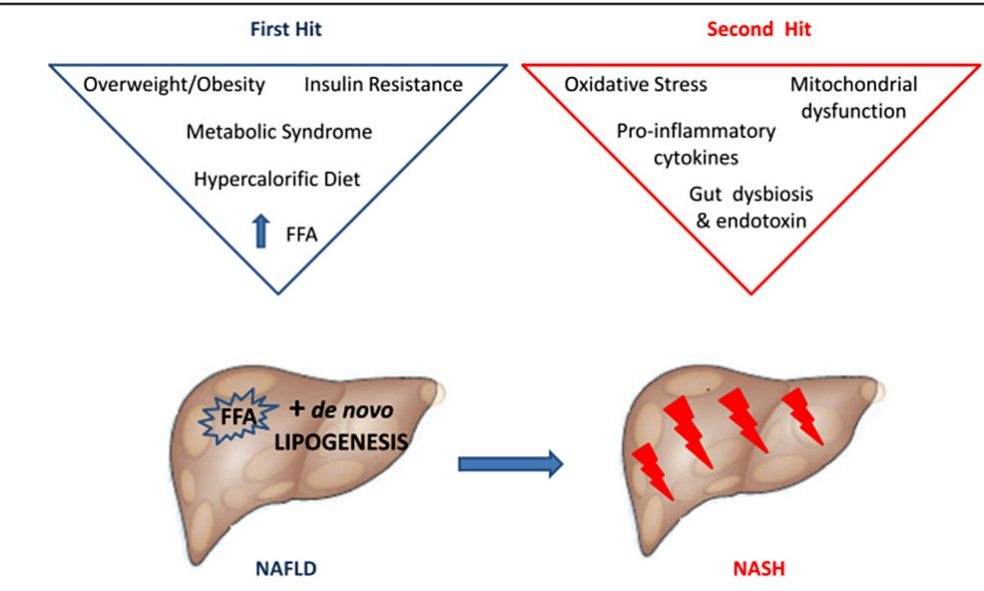
The pathogenesis of NAFLD and NASH NAFLD, non-alcoholic fatty liver disease; NASH, non-alcoholic steatohepatitis This figure is sourced from a journal [8]. This content is free to be copied and redistributed in any medium or format. The license is CC BY 2.0 and the figure has not been edited or changed.

Histologic evaluation in conjunction with liver biopsy is still the gold standard for diagnosing NAFLD. For NAFLD to be diagnosed, hepatic steatosis, ballooning, and lobular inflammation with or without fibrosis have to be present [4]. Grade 1 (mild), grade 2 (moderate), and grade 3 (severe) are used to categorize the necro-inflammatory grades of NASH [5,6]. Blood tests such as alanine transaminase (ALT) and aspartate aminotransferase (AST), which are supportive for diagnosing NAFLD, and other non-invasive techniques for diagnosing NAFLD including ultrasonography, computed tomography (CT) scans, and magnetic resonance imaging were done [7]. The pathogenesis of NAFLD and NASH is shown in Figure 1 [8].

Metabolic syndrome (MetS) is a group of metabolic abnormalities that includes hypertension, central obesity, insulin resistance, and dyslipidemia [9]. Adults in the United States over 18 continue to have a considerable prevalence of MetS. The incidence was reported to be 25.3% in the 1980s and rose to 34.2% in 2012. However, the most recent data from the National Health and Nutrition Examination Survey (NHANES) shows the prevalence is declining with 24% in men and 22% in women [10,11]. Around 70-80% of people with diabetes have been diagnosed with MetS, according to data, and it has been demonstrated to lower type 2 diabetic patients' survival rates by at least 10 years [12,13]. NAFLD was formerly thought to be a hepatic component of MetS, but more recently, a link between NAFLD and MetS in type 2 diabetes mellitus has been reported. NAFLD and MetS are, in fact, inversely and reciprocally correlated, with MetS serving as both its cause and its effect [12]. 

**Figure 2:**
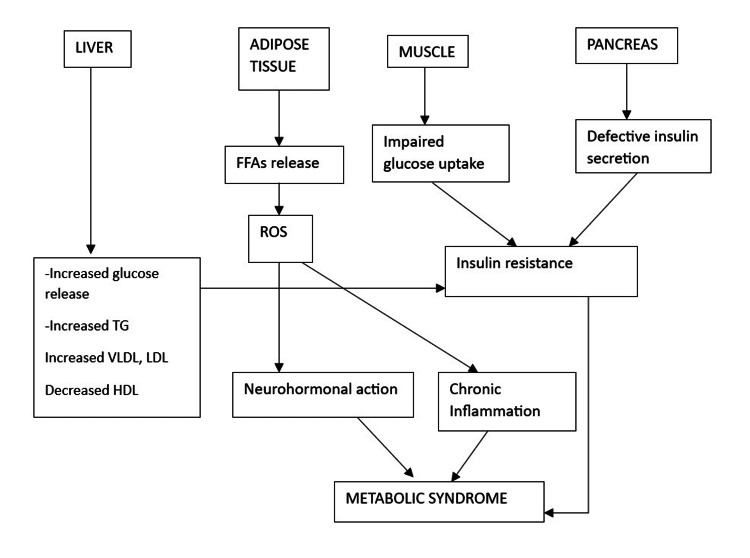
The etiology of MetS ROS, reactive oxygen species; TG, triglyceride; VLDL, very low-density lipoprotein; LDL, low-density lipoprotein; HDL, high-density lipoprotein; FFA, free fatty acid; MetS, metabolic syndrome Image credit: Dr. Zareen Zohara, the corresponding author of the current study.

The National Cholesterol Education Program Adult Treatment Panel III (NCEP/ATP-III) and criteria for the clinical diagnosis of metabolic syndrome (CCDMIA) were initially used to diagnose MetS [14,15]. The American Heart Association (AHA) and the National Heart, Lung, and Blood Institute (NHLBI) updated the ATP III criteria for diagnosis in 2005 [16]. Abdominal obesity (waist circumference >102 cm for men and >88 cm for women), elevated triglycerides (TGs) (>150 mg/dl or on drug treatment for elevated TGs), reduced HDL-C level (40 mg/dl in men, 50 mg/dl in women, or on drug treatment for reduced HDL-C), and hypertension (systolic blood pressure) are all indicators of the MetS. The pathophysiology of MetS includes several intricate mechanisms that are not fully understood. The question of whether the various MetS components constitute separate illnesses on their own or are part of a single, more general pathogenic process is still up for dispute. Overeating and a lack of physical activity have been identified as key contributors to the development of MetS, in addition to genetic and epigenetic variables. Since visceral adiposity is a significant trigger that activates most of the MetS pathways, high caloric intake can be attributed to a causal role. Figure 2 illustrates the etiology of MetS [9].

MetS and NAFLD frequently coexist in patients, supporting the well-established link between the two conditions [17]. Central obesity, steatosis, and insulin resistance are associated with NAFLD and MetS [18]. Due to the small sample populations, the strength of the evidence for a connection between NAFLD and MetS is questionable, despite growing experimental and epidemiological evidence suggesting both conditions coexist [19]. 

Independent of age, gender, and body mass index (BMI), Kotronen et al. found that people with MetS had considerably higher liver fat content than those without the disease [20]. On the other hand, it is less clear how MetS and NAFLD are related, with some research indicating that NAFLD functions as a precursor to MetS while others state the vice-versa [21-23]. Zhang et al. used a Bayesian network to investigate the reciprocal causation between MetS and NAFLD in a Chinese population to address these interactions. They found that the impact of MetS on NAFLD development was greater than the impact of NAFLD on MetS development [24]. According to estimates, NAFLD affects between 6.3% and 33% of the world's population, overall with prevalence rates among those who have metabolic co-morbidities being significantly greater [25]. Despite consistent advancements in understanding the etiology of NAFLD, discovering therapeutic targets, and expanding drug development, there are still many unanswered questions, and there are no authorized treatments for this condition yet. In addition to this, there is no awareness among the general public about the risk factors and development of this disease. However, the antecedent component has not been firmly established, mostly because of the limited research in this area, which has produced results that cannot be regarded as conclusive. For the above-mentioned reasons, we searched through the available literature to ascertain the relationship between NAFLD and MetS and to give a clear idea of the risk factors in this systematic research.

## Review

Methods

Reporting Guideline

This systematic research aims to identify the presence of NAFLD in patients with MetS. This systematic review has been conducted according to the Preferred Reporting Items for Systematic Reviews and Meta-Analyses (PRISMA) 2020 guidelines [26]. As secondary data from published articles were used, ethical approval was not considered.

Search Strategy

The search was conducted online on PubMed, Google Scholar, Science Direct, BMC, and Cochrane. The last search on all databases was on Feb 23, 2023. Keywords that have been utilized for the websites include MetS, syndrome X, insulin resistance, obesity, type 2 diabetes, and dyslipidemia. Keywords presented in this systematic review were targeted by Medical Subject Headings search (Mesh). The Boolean method was used to combine the keywords to create a uniform search through the various databases mentioned above. We identified 1106 potentially eligible records across all the databases. Different search strategies used in various journals have been summarized in Table 1.

**Table 1:** A summary of search strategies used in PubMed, Google Scholar, Science Direct, BMC, and Cochrane

Database	Keywords	Search Strategy	Filters Used	Results
PubMed	“Metabolic Syndrome,” “Syndrome X,” “Insulin resistance,” “Obesity,” “Type 2 diabetes,” “Dyslipidaemia,”	Metabolic Syndrome OR Syndrome X OR Insulin resistance OR Obesity OR Type 2 diabetes OR Dyslipidaemia OR ("Metabolic Syndrome/classification"(Mesh) OR "Metabolic Syndrome/diagnosis" (Mesh) OR "Metabolic Syndrome/diet therapy" (Mesh) OR "Metabolic Syndrome/etiology" (Mesh) OR "Metabolic Syndrome/genetics" (Mesh) OR "Metabolic Syndrome/immunology" (Mesh) OR "Metabolic Syndrome/metabolism" (Mesh) OR "Metabolic Syndrome/pathology" (Mesh) OR "Metabolic Syndrome/physiopathology" (Mesh) OR "Metabolic Syndrome/prevention and control" (Mesh) OR "Metabolic Syndrome/therapy" (Mesh) AND Non-Alcoholic fatty liver OR Non-Alcoholic steatohepatitis OR ("Non-alcoholic Fatty Liver Disease/diagnosis" (Mesh)) OR "Non-alcoholic Fatty Liver Disease/diet therapy" (Mesh) OR "Non-alcoholic Fatty Liver Disease/drug therapy" (Mesh) OR "Non-alcoholic Fatty Liver Disease/aetiology" (Mesh) OR "Non-alcoholic Fatty Liver Disease/genetics" (Mesh) OR "Non-alcoholic Fatty Liver Disease/immunology" (Mesh) OR "Non-alcoholic Fatty Liver Disease/metabolism" (Mesh) OR "Non-alcoholic Fatty Liver Disease/microbiology" (Mesh) OR "Non-alcoholic Fatty Liver Disease/pathology" (Mesh) OR "Non-alcoholic Fatty Liver Disease/physiopathology" (Mesh) OR "Non-alcoholic Fatty Liver Disease/prevention and control" (Mesh) OR "Non-alcoholic Fatty Liver Disease/therapy" (Mesh))	Published between 2010 and 2023	558
Google Scholar	“Metabolic Syndrome,” “Syndrome X,” “Insulin resistance,” “Obesity,” “Type 2 diabetes,” “Dyslipidaemia.”	Metabolic syndrome, non-alcoholic fatty liver disease	Advanced search with all the keywords only in the title	537
Science Direct	“Metabolic Syndrome,” “Syndrome X,” “Insulin resistance,” “Obesity,” “Type 2 diabetes,” “Dyslipidaemia.”	Metabolic syndrome, non-alcoholic fatty liver disease	No filters used	7
BMC	“Metabolic Syndrome,” “Syndrome X,” “Insulin resistance,” “Obesity,” “Type 2 diabetes,” “Dyslipidaemia.”	Metabolic syndrome, Non-alcoholic fatty liver disease	No filters used	3
Cochrane	“Metabolic Syndrome,” “Syndrome X,” “Insulin resistance,” “Obesity,” “Type 2 diabetes,” “Dyslipidaemia.”	Metabolic syndrome, Non-alcoholic fatty liver disease	No filters used	1

Eligibility Criteria

The studies that were selected are based on the participants and outcomes. Participants are the studies with young adults (19-21), early adults (22-34), early middle age (34-44), and late middle age (45-64) from all ethnicities and genders with MetS with or without a diagnosed NAFLD. The outcomes are studies showing evidence of the increased frequency of NAFLD in MetS patients.

Inclusion Criteria

The inclusion criteria in this systematic review included studies that compare NAFLD and MetS. There were no gender or ethnic restrictions for the population of the studies. We restricted our search to online records issued in English, available as free full texts, including human participants, and issued from Feb 23, 2010, to Feb 23, 2023. In addition, randomized controlled trials (RCTs), observational studies, meta-analyses, literature, and systematic reviews were included in the study.

Exclusion Criteria

We did not include articles written in languages other than English, studies done in animals or patients younger than 19 years old, case reports, unpublished articles, editorials, or articles done before 2010. Population, intervention, comparison, and outcomes (PICO) criteria were used as a framework for our eligibility criteria.

Data Selection and Extraction

Two distinct authors (the first and second authors) independently selected and extracted the relevant studies. The two researchers resolved disagreements over eligibility by discussing the study design, intervention implemented, outcomes measured, and the relevance to our inclusion and exclusion criteria. Through the five databases, 995 articles were selected after EndNote (Clarivate, Philadelphia, PA) software removed duplicates. We applied the inclusion and exclusion criteria with the search strategy at the beginning of the screening and identified articles directly related to the topic. The researchers analyzed the articles during the screening process, and 37 articles were selected for quality appraisal, of which 24 were cross-sectional, four were case-control, and seven articles were from review, along with two articles from grey literature. These 37 were screened for quality appraisal.

Quality Assessment

The 37 chosen articles are then assessed by two authors (the first and second authors) using tools such as Newcastle-Ottawa Scale adapted for cohort and case-control studies and the SANRA (scale for the assessment of narrative review articles) checklist for review articles [27,28]. Two authors investigated the risk of bias using tools. Each study was assessed by these tools and scored accordingly. Studies with a minimum accepted scoring of >70% in checklists were selected. This selection has been briefly summarized in Table 2 [29-43].

**Table 2:** The selected articles after quality appraisal SANRA, the scale for the assessment of narrative review articles

Studies Approved After Review	Study Type	Quality Appraisal Tool	The Total Score of the Tool	Acceptable Score
Agrawal PK et al. [29]	Cross-sectional study	Newcastle-Ottawa scale	9	7
Fattahi MR et al. [30]	Cross-sectional study	Newcastle-Ottawa scale	9	7
Goyal A, Arora H, Arora S [31]	Cross-sectional study	Newcastle-Ottawa scale	9	7
Asati P, Kukrele P, Jalodiya S [32]	Cross-sectional study	Newcastle-Ottawa scale	9	7
Onyekwere CA, Ogbera AO, Balogun BO [33]	Cross-sectional study	Newcastle-Ottawa scale	9	7
Godoy-Matos AF, Silva Júnior WS, Valerio CM [34]	Review article	SANRA	12	9
Iftikhar R et al. [35]	Cross-sectional study	Newcastle-Ottawa scale	9	7
Ratnasari N et al. [36]	Case-control study	Newcastle-Ottawa scale	8	5
Gavril OI et al. [37]	Cross-sectional study	Newcastle-Ottawa scale	9	7
Pardhe BD et al. [38]	Cross-sectional study	Newcastle-Ottawa scale	9	7
Paudel MS et al. [39]	Cross-sectional study	Newcastle-Ottawa scale	9	7
Suppiah S et al. [40]	Cross-sectional study	Newcastle-Ottawa scale	9	7
Yuan Q et al. [41]	Cross-sectional study	Newcastle-Ottawa scale	9	7
Souza MR et al. [42]	Review article	SANRA	12	9
Zakerkish M et al. [43]	Cross-sectional study	Newcastle-Ottawa scale	9	7

Results

**Figure 3:**
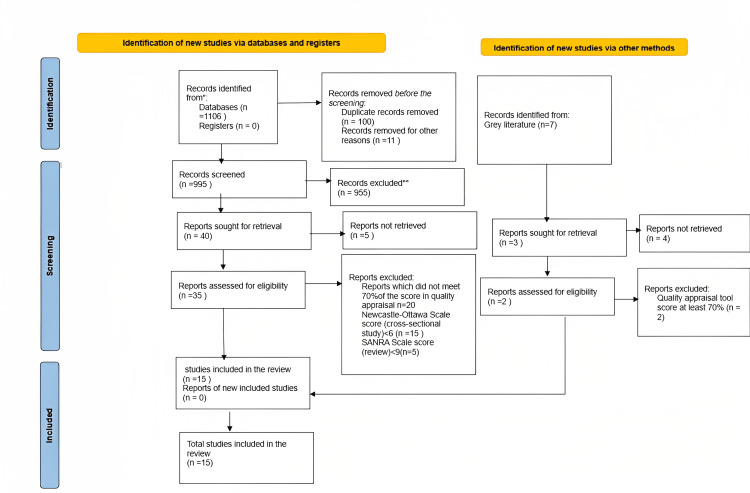
Flowchart of the PRISMA PRISMA: Preferred Reporting Items for Systematic Reviews and Meta-Analyses.

All five databases underwent screening for groups with MetS and NAFLD. Initially, we found 203059 papers based on the search results. After applying several filters such as the English language, free-full text, keywords in the title, and our inclusion/exclusion criteria, we reduced our search to 1106 papers. A hundred duplicates were eliminated by EndNote (Clarivate, Philadelphia, PA) software before the studies were screened and chosen. The 995 articles underwent a rigorous review to determine whether the titles and abstracts were pertinent to our review. 955 of the articles screened were irrelevant to the topic, goals, inclusion, or exclusion criteria, and were eliminated. Consequently, 35 articles were chosen for quality appraisal. Additionally, seven articles were identified from grey literature, and two were assessed for quality appraisal. 37 papers were ultimately screened for quality appraisal and eligibility check. The PRISMA flowchart depicts this process in detail in Figure 3.

A total of 15 articles made it to the final review [29-43]. Table 3 contains the details of the relevant 15 articles included [29-43].

**Table 3:** Details of the relevant 15 articles after quality appraisal NAFLD, non-alcoholic fatty liver disease; MetS, metabolic syndrome; NCEP, National Cholesterol Education Program; ATP, adult treatment panel; ALT, alanine transaminase; AST, aspartate aminotransferase; BMI, body mass index; HDL, high-density lipoprotein; FFA, free fatty acid; CT, computed tomography

Serial number	Author	year	Type	Purpose	Result	Conclusion
1	Agrawal P K et al. [29]	2017	Cross-sectional	This study was conducted to look into the clinical profile of patients of NAFLD and evaluate the relationship between NAFLD and MetS as defined by the modified NCEP ATP III criteria.	The prevalence of NAFLD was found to be 18.78% and was higher among the male population (20.05%) than females (17.32%). The prevalence of MetS among NAFLD is 42.74%.	The present study has shown a moderate prevalence of NAFLD and MetS among the rural population of western Uttar Pradesh with a more male predisposition.
2	Fattahi MR et al. [30]	2016	Cross-sectional	Growing experimental and epidemiological evidence suggests that NAFLD and MetS share common interactions. However, the definite evidence of a link between NAFLD and MetS is uncertain due to the small study populations.14 We analyzed the prevalence of NAFLD and MetS in a large population.	MetS was detected in 65.9% and 64.6% of the patients with NAFLD (based on NCEP/ATP-III) and in 30.1% and 73.7% (based on CCDMIA) of men and women, respectively. None of the components had significant differences between the two genders (p>0.05). Although the odds ratio for hyperglycemia and abdominal obesity were approximately high in CCD MIA criteria (0.9613 and 1.2082, respectively), the differences were not statistically significant.	NAFLD was associated with MetS. However, it was not possible to determine whether NAFLD predates the development of MetS.
3	Goyal A et al. [31]	2020	Cross-sectional	To research the frequency of NAFLD in the MetS and the relationship between NAFLD and the syndrome's elements.	Out of 52 hypertensive patients, 37 (71%) had fatty liver. Among non-hypertensive cases, 36 (75%) had fatty liver.	MetS was discovered to have a high prevalence of fatty liver, and early diagnosis of fatty liver can alter the course of the disease and postpone more severe problems such as liver cirrhosis and hepatocellular cancer.
4	Asati P et al. [32]	2017	Cross-sectional	To examine the clinical characteristics of individuals with NAFLD who have been given an ultrasonographic diagnosis and to assess the connection between NAFLD and MetS and its elements.	51% of patients with NAFLD had Mets, and statistical significance was found in AST, waist circumference, lipid profile total cholesterol was significantly higher in grade 3 NAFLD which was significantly higher. AST and ALT values were significantly higher among grade 3 NAFLD cases.	Our study reveals that there is a higher prevalence of all the components of MetS in cases of NAFLD.
5	Onyekwere CA et al. [33]	2011	Cross-sectional	To determine the prevalence of NAFLD among a population of diabetic subjects attending the endocrine clinic of LASUTH compared with non-diabetic subjects; ascertain other contributing factors and compare the occurrence of the MetS in subjects with and without NAFLD.	150 subjects, with a mean age of 56 years (standard deviation=9, range 20-80 years) and a gender ratio (F:M) of 83:67(55%:45%) were recruited. 106 were diabetics, and 44 were non-diabetics. The overall prevalence of NAFLD amongst all study subjects was 8.7%. The prevalence rate of NAFLD was higher in the diabetes cases than in the control subjects, but this difference was not statistically significant (9.5 vs. 4.5%, p=0.2).	NAFLD is present in Africa but is less than what one would expect based on American and European studies.
6	Godoy-Matos AF et al. [34]	2020	Review	This review will focus on the clinical and pathophysiological connections between NAFLD, insulin resistance, and type 2 diabetes.	The NAFLD spectrum is correctly understood as a continuum from obesity to MetS and diabetes.	The development of tailored treatment and early identification may benefit from a proper understanding of the NAFLD spectrum, which is best understood as a continuum from obesity to MetS and diabetes.
7	Iftikhar R et al. [35]	2015	Cross-sectional	To determine the frequency of NAFLD disease in patients with MetS.	Of 491 participants with MetS, 222 (45.2%) had fatty liver disease. The mean BMI in patients with Mets was 26.1 (± .89), and the mean BMI in fatty liver patients was 27.3 (± 0.67).	A large number of patients with MetS have fatty liver disease. Fatty liver disease is more frequent in patients who are overweight and those having multiple risk factors for MetS.
8	Ratnasari N et al. [36]	2012	Case-control	This study aimed to know the risk factors of NAFLD related to MetS.	There were 84 patients enrolled in the study (group I=30 NAFLD+MetS subjects; group II=26 NAFLD patients; group III=28 healthy). The data showed statistical significance.	Systolic blood pressure, fasting glucose, triglyceride, HDL, cholesterol, FFA, and adiponectin are significant factors of NAFLD prevalence in MetS. However, independent factors that were statistically significant were only FFA and HDL cholesterol.
9	Gavril OI et al. [37]	2019	Cross-sectional	This study aimed to evaluate, among diabetic subjects, the relationship between fatty liver load and the presence of MetS criteria.	Most of the patients included in the study had varying degrees of liver fat load and met the criteria for MetS (81,31%). It was found that the frequency of cases with fatty liver impairment was significantly higher in subjects with MetS (32,43% compared to 5,88% for those without Mets).	We can say that NAFLD is a risk factor for the presence of MetS and it can be considered the hepatic expression of this syndrome.
10	Pardhe BD et al. [38]	2018	Cross-sectional	This study aims to recognize various risk factors including metabolic components and blood parameters to predict the possible incidence of the disease.	Further, according to the NCEP ATP III criteria, 13.6% of NAFLD were present with MetS where the risk estimate was significant. Whereas, other criteria for MetS showed higher frequency (30.1%) with higher risk for the presence of MetS in NAFLD patients.	The result of this study suggests that there is an increased prevalence of all the components of MetS and significant changes in biochemical markers in cases of NAFLD. Timely diagnosis would help in delaying its complications and co-morbidities.
11	Paudel MS et al. [39]	2019	Cross-sectional	This study aims to determine the prevalence of MetS in Nepalese patients with NAFLD from the mid-Western part of Nepal.	A total of 385 participants with NAFLD were evaluated. The presence of MetS by NCEP-ATP III criteria was found in 57.6% of participants; whereas, at least one component of MetS was found in 91.4% of participants with radiologic features of fatty liver.	MetS is common in Nepalese community patients with NAFLD.
12	Suppiah S et al. [40]	2016	Cross-sectional	Our goal was to figure out how common NAFLD was among patients with MetS.	In our population, 82.8% of individuals with MetS had NAFLD. The older population has a significant NAFLD prevalence.	We do not advise using CT as a sole, initial inquiry to treat NAFLD in those with MetS.
13	Yuan Q et al. [41]	2022	Cross-sectional	The goal of this study was to estimate the prevalence and risk factors of NAFLD among Beijing adults aged ≥25 years old.	The prevalence of NAFLD was 32.40%. Risk factors independently associated with NAFLD included male gender urban residence, older age, and lower education middle school. NAFLD was more common in females than in males after 50 years of age. Lean/normal weight NAFLD patients account for approximately 3.04% of NAFLD	Compared to non-NAFLD subjects, the lean/normal NAFLD patients had a higher prevalence of hypertension and diabetes and a higher degree of hepatic steatosis and liver function.
14	Souza MR et al. [42]	2012	Review	To review the literature about the major risk factors for NAFLD in the context of MetS, focusing on underlying mechanisms and prevention.	The final analysis of all these data, pointed out central obesity, type 2 diabetes, dyslipidemia, and hypertension as the best risk factors related to NAFLD.	Risk factors for NAFLD in the context of MetS expands the prospects to recognize patients with MetS at high risk for NAFLD, elucidate pathways common to other co-morbidities, determine risk factors associated with a worse prognosis, develop therapeutic strategies to reduce risk factors, apply acquired knowledge in public health policies focusing on preventive strategies.
15	Zakerkish M et al [43]	2021	Cross-sectional study	This study was to determine the prevalence of MetS in patients with NAFLD.	MetS were present in 63.84% of NAFLD patients.	The current study's findings revealed that a significant proportion of NAFLD is with MetS.

Discussion

NAFLD is one of the drastically developing diseases in the global population. This research focuses on the relationship between NAFLD and MetS, its risk factors, and its association with demographic factors. This includes the relationship between the components of MetS, such as visceral obesity, dyslipidemia, hyperglycemia, hypertension, and non-alcoholic fatty liver, along with the demographic variables like age, gender, ethnicity, educational attainment, source of income, and socio-economic status. Many publications released in the last few years have demonstrated only the relationship between NAFLD and MetS. The studies evaluated in this systematic review showed multiple aspects and outcomes regarding the risk factors underlying the development of NAFLD in MetS.

Relationship Between NAFLD and Metabolic Syndrome

In studies that find the association between NAFLD and MetS, the prevalence of NAFLD in MetS is found to be higher (91.4%) [29,31,32,37,39,43,40]. The above-mentioned studies have found a statistically significant association between NAFLD and MetS in study participants from India, Iran, Malaysia, Nepal, and China. These studies identified the cases of NAFLD by using ultrasonography and CT, while identifing MetS by NCEP: ATP III criteria [29,31,32,40,43]. NAFLD was significantly more common in people with MetS (43.2%) and increased with the number of MetS criteria (67% for those with all five criteria), despite the prevalence of NAFLD being 18.2%. Furthermore, advanced liver fibrosis was seen in 6.6% of people with moderate to severe steatosis, almost doubled in people with MetS, and impressively reached 30% in people with five MetS criteria [34]. While these studies find a positive relationship between NAFLD and MetS, Fattahi et al. study demonstrated that NAFLD was associated with MetS, and with their analysis, it was not possible to determine whether NAFLD predates the development of MetS [30]. Oana et al. study illustrated that NAFLD is a risk factor for the presence of MetS, and it can be considered the hepatic expression of this syndrome [37].

Demographic Variants in the Development of NAFLD and MetS

In the attempt to find the relationship between the various demographic variants, certain study shows that the prevalence of NAFLD among MetS is high among male than female population, and males are more susceptible to grade 2 fatty livers, while women are more susceptible to grade 1 [29,43]. The prevalence of NAFLD in males peaked at 40-49 years and then began to decline. NAFLD was more common in females than in males after 50 years of age, while there is no significance in other studies [31,32,37,40,41]. The prevalence of MetS in this research population varied depending on the diagnostic standard utilized (NCEP/ATP-III or CCDMIA). When the NCEP/ATP-III criteria were applied, the prevalence of MetS tended to be higher in men, and when the CCDMIA was applied, it tended to be higher in women [30].

Concerning the population's age, the elderly age group (57 years old and above) had the highest prevalence of NAFLD, while a study conducted in India shows no association with age [31,40,41]. Regarding the population's distribution, 59% of the cases were from rural backgrounds, 41% from urban backgrounds, and 72% and 73%, respectively, of the cases, had fatty livers [31]. The prevalence of NAFLD is comparatively higher among urban people. Their greater likelihood of having bad eating habits and overnutrition issues, which may result in fat accumulation, may be explained by their higher life quality, and considering the population's ethnicity, the overall prevalence of NAFLD is highest among Malay ethnics with 79.7% of MetS X Malays being detected with NAFLD [40,41]. Relating to the population's level of education, middle school graduates had the highest rate of NAFLD. Education level influences the recognized prevalence of NAFLD knowledge of onset and prevention. Therefore, individuals with higher levels of education may have fewer negative variables like eating disorders and obesity [41].

Risk Factors in the Development of NAFLD

The risk factors related to NAFLD are studied along with the attempt to find the most prevalent risk factor in this systematic review and it revealed that in a study the incidence of MetS, which includes NAFLD, is rising in India due to the country's rising obesity rate, and the MetS itself is now considered a risk factor for NAFLD [29,32]. Obese patients have demonstrated the role of adipocytokines in the etiopathogenesis of NAFLD. As compared to both obese and non-obese groups, NAFLD patients had a considerable increase in tumor necrosis factor-alpha (TNF-alpha), interleukin-8 (IL-8), and lower adiponectin levels [36]. It is postulated that the increased prevalence of insulin resistance and subsequently fatty liver in Asian-Indian men was associated with increased levels of interleukin-6 concentrations, a mediator of inflammation [40]. There is a positive association between obesity and NAFLD [31,35,36,38,39]. Patients with central obesity are typically more insulin resistant and more likely to have NAFLD than those with lower-body obesity because the central obesity phenotype is linked to increased intra-abdominal fat [42]. Apart from this, hyperglycemia is one of the components of MetS. Its correlation with liver disease has been studied in various studies, and it found a high prevalence of hyperglycemia in the ultrasound-confirmed liver disease group than in the participants without the liver disease [31,35,38,39,42,43]. The other component of MetS is hypertension, of which hypertension and NAFLD are positively correlated, according to studies [31,35,38,39,42,43]. Moreover, there was no correlation between NAFLD and hyperlipidemia in the subset of our research with type 2 diabetes mellitus. This may be because diabetes mellitus is not the primary cause of fatty liver in patients with dyslipidemia. For example, dyslipidemia is a separate component in the development of NAFLD. The prevalence of hypertension and MetS steadily rose along with the NAFLD's rising BMI [41]. Above all, in studies, 438 soldiers with MetS (89%) were determined to be physically inactive, and 213 of them (48.6%) had fatty livers [35,42].

Biochemical Parameters Associated with NAFLD and MetS

Various blood parametric changes are found to be associated with NAFLD and MetS, which have been analyzed in various studies. Almost all the study showed changes in the blood parameters like total cholesterol, TG, ALT, AST, high-density lipoprotein (HDL), very low-density lipoprotein (VLDL), low-density lipoprotein (LDL), and fasting blood sugar (FBS). There is a statistically significant association with fasting glucose, TG, HDL-C, (homeostasis model assessment-estimated insulin resistance) HOMA index ratio, and adiponectin [36]. Moreover, ALT changes between NAFLD grades were substantial although AST changes between grades were not [38,41]. The TG was found to rise and HDL-C to fall with steatosis [37,38]. Moreover, it was found that there is a rise in hypertriglyceridemia, low HDL, hypercholesterolemia, LDL, and ALT [31,35,37,39,40,41,43].

Given the aforementioned, the findings from studies can be summarized that NAFLD is predominant in MetS patients. The preceding dilemma is not known earlier, but now it is evident that MetS is a definite risk factor for NAFLD, and even one component of MetS is associated with NAFLD. Moreover, demographic variants play a major role as a deciding factor in the development of NAFLD. NAFLD is more prevalent in the elderly with an increasing frequency in females after age 50. This can be explained by the estrogenic action, which ultimately proves the relationship between obesity and NAFLD. In the bargain, the population distribution, economic status, and education impact the development of NAFLD. Regarding risk factors, obesity plays a major part in the pathogenesis of NAFLD development, which is evident from the fact that NAFLD is more predominant in elderly females. The findings suggested that low estrogen levels throughout the postmenopausal period may be a significant risk factor for female NAFLD in old age by preventing the accumulation of peripheral gluteal-femoral subcutaneous adipose tissue within the liver. It is also clear from various studies that although dyslipidemia and diabetes do not coexist in most cases, dyslipidemia alone can be postulated as a threat to NAFLD. Dyslipidemia acts as an independent component in the development of NAFLD. Likewise, insulin resistance is the key factor for the development of this. Besides various demographic and risk factors, physical activity and diet also play a role in the development of NAFLD. It is found that patients with NAFLD show an increased level of ALT, AST, LDL, TC, FBS, and low HDL levels, which can be an alarming tool for NAFLD.

Limitations

Several limitations were found in our systematic review. The first restriction is provided by the standards established as filters for screening papers. The number of resources is decreased by using only free full-text articles in English and by including human studies between 2010 and 2023. Second, a variety of study types, including observational, randomized clinical trials, systematic reviews, and meta-analyses, were chosen. As a result, the samples and statistical techniques varied, which leaves our revision vulnerable to bias. Third, due to the high complexity of the disease, several studies were conducted with small samples. In this situation, it is crucial to emphasize that studies with larger samples are preferable to acquire more conclusive results to ensure the presence of MetS as a precursor.

## Conclusions

With the increasing emergence of NAFLD as a threat to the world population, an attempt is made to analyze the relationship between NAFLD and MetS in finding the preceding component. As analyzed from the data, NAFLD is more common in the group of people with MetS. The predicament was unknown earlier, but it is now obvious that MetS is a known risk factor for NAFLD. The pathophysiology and insulin resistance that both disorders share, together with the fact that obesity is the root of both, serve as the connecting elements for NAFLD and MetS. Additionally, demographic differences, age, population distribution, economic standing, and educational attainment all play a role in the development of NAFLD. Regarding risk factors, obesity and dyslipidemia is a significant contributor to the pathogenesis of NAFLD. Physical activity and food, in addition to several demographic and risk variables, also affect the development of NAFLD. Additional recommendations include supervising and piloting more studies, particularly cohorts and RCTs with larger samples; combination medicines; extended follow-up periods; and the effect of diet and exercise in preventing, curing, and managing the disease. These concepts are brought up to help readers understand the associated risk factors, which demographic factor is more prevalent and to enlighten the population about lifestyle changes being one of the important risk factors for the disease and potentially to fill in any gaps and inconsistencies discovered during this systematic review. To improve and elaborate future evidence, it is vital to note that further research on the development of NAFLD is required. It is also recommended to work on finding advanced techniques for managing this disease. It is also recommended to work further on the dietary changes that would help in the management of this disease. 
